# Transcutaneous induction of stimulus-timing-dependent plasticity in dorsal cochlear nucleus

**DOI:** 10.3389/fnsys.2015.00116

**Published:** 2015-08-14

**Authors:** Calvin Wu, David T. Martel, Susan E. Shore

**Affiliations:** ^1^Kresge Hearing Research Institute–Department of Otolaryngology, University of MichiganAnn Arbor, MI, USA; ^2^Department of Biomedical Engineering, University of MichiganAnn Arbor, MI, USA; ^3^Department of Molecular and Integrative Physiology, University of MichiganAnn Arbor, MI, USA

**Keywords:** transcutaneous electrical nerve stimulation, stimulus-timing-dependent plasticity, tinnitus, dorsal cochlear nucleus, multisensory integration

## Abstract

The cochlear nucleus (CN) is the first site of multisensory integration in the ascending auditory pathway. The principal output neurons of the dorsal cochlear nucleus (DCN), fusiform cells, receive somatosensory information relayed by the CN granule cells from the trigeminal and dorsal column pathways. Integration of somatosensory and auditory inputs results in long-term enhancement or suppression in a stimulus-timing-dependent manner. Here, we demonstrate that stimulus-timing-dependent plasticity (STDP) can be induced in DCN fusiform cells using paired auditory and transcutaneous electrical stimulation of the face and neck to activate trigeminal and dorsal column pathways to the CN, respectively. Long-lasting changes in fusiform cell firing rates persisted for up to 2 h after this bimodal stimulation, and followed Hebbian or anti-Hebbian rules, depending on tone duration, but not somatosensory stimulation location: 50 ms paired tones evoked predominantly Hebbian, while 10 ms paired tones evoked predominantly anti-Hebbian plasticity. The tone-duration-dependent STDP was strongly correlated with first inter-spike intervals, implicating intrinsic cellular properties as determinants of STDP. This study demonstrates that transcutaneous stimulation with precise auditory–somatosensory timing parameters can non-invasively induce fusiform cell long-term modulation, which could be harnessed in the future to moderate tinnitus-related hyperactivity in DCN.

## Introduction

The cochlear nucleus (CN) receives auditory nerve fiber (ANF) inputs from the cochlea, as well as projections from somatosensory afferents. The trigeminal and dorsal column pathways send axonal terminals to the marginal area of the ventral cochlear nucleus (VCN) and the small cell cap, collectively defined as the granule cell domain (GCD; Wright and Ryugo, 1996; Shore et al., 2000; Zhou and Shore, 2004; Zhan et al., 2006; Zeng et al., 2011). CN granule cell axons relay somatosensory inputs to the apical dendrites of fusiform cells and associated inhibitory interneurons (Davis et al., 1996; Davis and Young, 1997; Golding and Oertel, 1997). Electrically stimulating the trigeminal ganglion or the spinal trigeminal nucleus (Sp5), cervical nerve or dorsal column brainstem nuclei can suppress or enhance fusiform cell responses to auditory stimuli (Kanold and Young, 2001; Shore, 2005; Kanold et al., 2011; Koehler et al., 2011; Dehmel et al., 2012).

Whether enhancement or suppression occurs is determined by the temporal order and interval of the combined auditory and somatosensory stimuli (Koehler and Shore, 2013a,b), which is mediated by spike-timing-dependent plasticity (sTDP) of the parallel fiber–fusiform cell synapse *in vitro* (Tzounopoulos et al., 2004, 2007). Fusiform cells show “Hebbian plasticity” when presynaptic potentials preceding post-synaptic spikes induce enhancement, while those following spikes induce suppression. Using tone stimulation to generate post-synaptic fusiform cell spikes and somatosensory stimulation to generate pre-synaptic potentials, the macromolecular correlate of sTDP, stimulus-timing-dependent plasticity (STDP), can be observed (Koehler and Shore, 2013a,b). STDP is a long-lasting process that likely mediates circuit formation and adaptive filtering of internal vs. external auditory cues (Bell et al., 1997; Nelson, 2004; Oertel and Young, 2004; Requarth and Sawtell, 2011). Maladaptive STDP in DCN occurs in tinnitus (Koehler and Shore, 2013a), the phantom perception of sound, which is characterized by fusiform cell hyperactivity (Brozoski et al., 2002; Kaltenbach et al., 2004, 2005; Dehmel et al., 2012; Koehler and Shore, 2013a).

In this study, we show that STDP can be induced by pairing auditory with transcutaneous activation of somatosensory pathways to the CN. Electrical stimulation of the trigeminal (face) and the dorsal column (neck) afferent pathways produced robust Hebbian or anti-Hebbian plasticity in fusiform cells. STDP was dependent on tone duration and associated with intrinsic properties of fusiform cells. These findings demonstrate a non-invasive approach to control DCN activity, providing feasibility for an effective and accessible tinnitus treatment strategy.

## Materials and Methods

### Surgical Preparation and Recording

All animal procedures were performed in accordance with protocols established by the National Institute of Health publication No. 80-23 and approved by the University Committee on Use and Care of animals at University of Michigan. Guinea pigs (*n* = 11; 326–985 g; Elm Hill Labs) were anesthetized subcutaneously (40 mg/kg ketamine – Putney Inc., 10 mg/kg xylazine—Lloyd Inc.) and secured with hollow ear bars in a stereotaxic frame (Kopf). Body temperature was kept constant at 38°C with a custom built encapsulating thermal pad and rectal probe. Anesthetic depth was assessed by hind leg withdrawal areflexia and maintained with hourly 10 mg/kg ketamine and 3 mg/kg xylazine injections. Incision sites were treated with topical lidocaine. Recordings were performed in a double walled sound-proof booth. Auditory brainstem responses were assessed prior to surgery to establish normal hearing thresholds. After a small craniotomy, a two-shank 16-channel recording probe (NeuroNexus) was placed stereotaxically into DCN through an intact cerebellum at a 25° angle from vertical, 4 mm caudal of interaural line, 3 mm lateral of midline, and a depth of 6–7 mm. Broadband noise bursts (65 dB SPL, 50 ms duration, 2 ms linear ramp rise/fall time) were used to locate units. Receptive fields (100–24 kHz tone bursts in 0.15 octave steps; 0–90 dB in 5 dB steps; 50 ms duration, 2 ms linear ramp rise/fall time) were recorded to determine thresholds and best frequencies (BFs). A suitable electrode location in the DCN fusiform cell layer was confirmed by robust responses to BF tones with buildup or pauser-buildup temporal patterns and type III tuning properties that are typical of DCN fusiform cells (Stabler et al., 1996). Animals were terminated with sodium pentobarbital at the end of each experiment.

### Transcutaneous Electrical Stimulation

**FIGURE 1:**
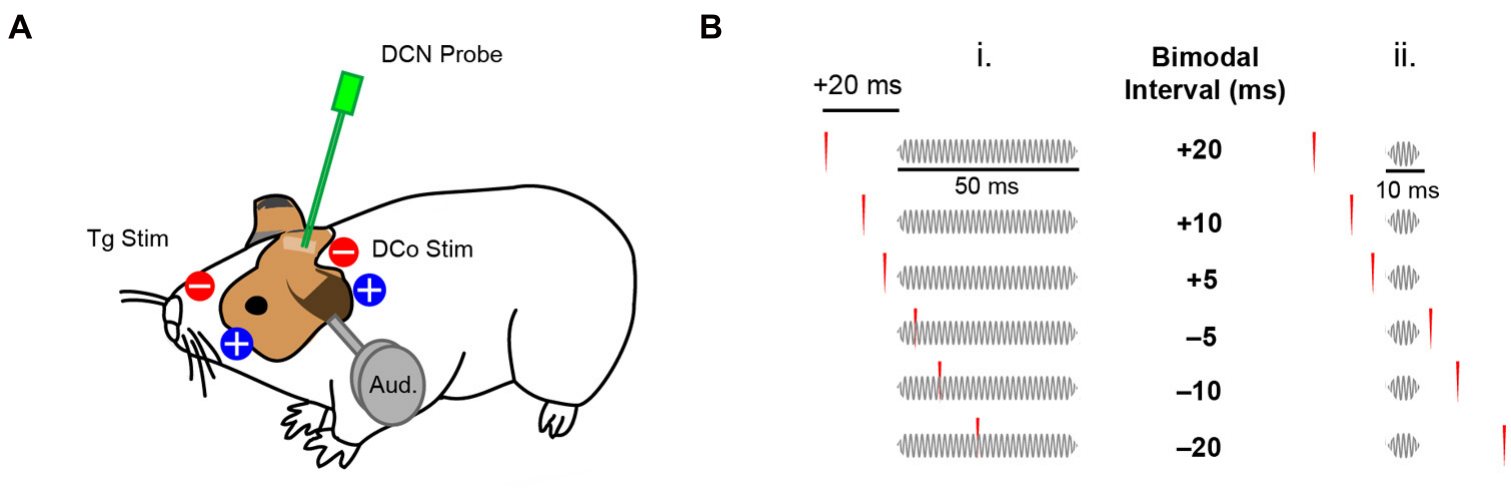
**Transcutaneous electrical stimulation and bimodal pairing protocol. (A)** Skin electrode pads were placed on the ipsilateral face and neck to activate the trigeminal (Tg) and dorsal column (DCo) somatosensory pathways. DCN neural responses were recorded during electrical and auditory stimulation (Aud). **(B)** Bimodal pairing using 50 ms (i) or 10 ms (ii) tones in a close temporal proximity with transcutaneous electrical stimulation. Positive bimodal intervals indicate preceding electrical stimulation while negative intervals indicate preceding auditory stimulation.

Animals were shaved in regions of transcutaneous electrode placement: for trigeminal pathway stimulation, an electrode pad (10 mm diameter Ag/AgCl Brainet electrode, Rhythmlink) was placed on the skin at the center of the left cheek superficial to the masseter muscle, with the ground electrode pad on the nasal bridge (**Figure 1A**). For dorsal column pathway stimulation, the electrode pad was placed on the neck 1 cm caudal to the ridge of the occipital bone and 1 cm lateral (left) to the midline. A ground electrode was placed 1 cm medial to the active electrode (**Figure 1A**). Three biphasic (100 μs/phase) current pulses at 1000 Hz (2.2 ms total duration) were delivered per trial at levels that evoked fusiform cell spikes above spontaneous rate (2–5 mA).

### Assessment of STDP

To assess STDP, 40 dB spike latency (SL) tones at BF (50 ms duration, 2 ms linear ramp rise/fall time, 100 repetitions at 5 per second) were presented before bimodal (auditory–somatosensory) pairing. 300 repetitions (5 per second) of either 50 ms or 10 ms duration tones (2 ms linear ramp rise/fall time) were paired with transcutaneous electrical stimulation of either trigeminal or dorsal column pathways with different bimodal intervals (temporal gap of electrical pulses relative to the tones). Bimodal intervals were either positive (electrical-preceding) or negative (auditory-preceding; **Figure 1B**). Responses to bimodal stimuli were compared to responses to the 40 dB SL BF tones alone, presented before, 5 and 15 min after bimodal stimulation to assess long-term changes in fusiform cell firing rates. In some experiments, persistent effects were assessed for up to 60 or 120 min to determine recovery. Persistent effects were quantified as percent changes in firing rate from the control, pre-bimodal tone alone condition. Recovery was defined as return to within ±10% of the pre-bimodal baseline at the earliest assessment time point after maximum bimodal enhancement or suppression. As a control, bimodal pairing was replaced with unimodal auditory or unimodal electrical stimulation. Bimodal intervals (-20, -10, -5, 5, 10, 20 ms), and unimodal auditory and electrical stimulation were randomized in each experiment. In some experiments, only one bimodal interval was used to control for potential effects of repeated stimuli.

### Data Analysis

Voltages recorded from multi-channel recording probes were digitized by a PZ2 (Tucker Davis Technologies) preamplifier and band-pass filtered (300–3 kHz) for spike detection: threshold was set at 2.5 SD above background noise. Recorded spike waveforms were sorted by principal components of the waveform shape and cluster analyses (Plexon Oﬄine Sorter). Electrical artifacts were identified by their distinct clusters and removed from further analysis. Spike waveforms remained consistent over the recording duration. Sorted spikes were imported to MATLAB as timestamps. Spike latencies were calculated using the bin-less Poisson method described by Chase and Young (2007). STDPs were presented as percent changes in spike rate as a function of bimodal interval, and classified into Hebbian or anti-Hebbian types. Other types of STDP such as enhancement or suppression reported previously *in vivo* (Koehler and Shore, 2013b) were not considered in further analysis. STDP indices (sums of relative changes for positive bimodal intervals subtracted from sums of relative changes for negative intervals) were computed to quantify Hebbian or anti-Hebbian tendencies. Statistical analyses were performed using the MATLAB statistical toolbox. Two-way analysis of variance (ANOVA), as well as non-parametric statistical tests for unequal population distributions were used. *Post hoc* tests for ANOVAs were conducted using the Tukey–Kramer method. Significance was established at α = 0.05.

## Results

### Transcutaneous Trigeminal and Dorsal Column Somatosensory Stimulation Activate Distinct Pathways to DCN

**FIGURE 2:**
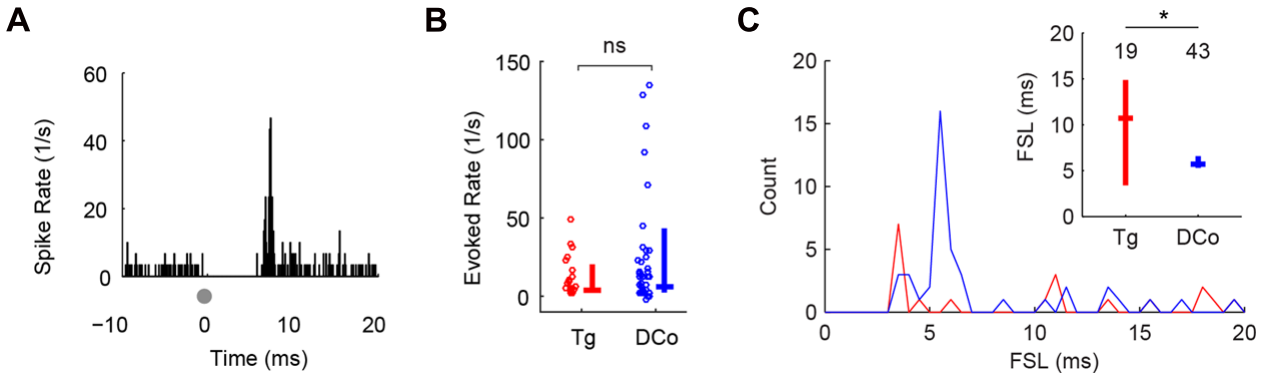
**Transcutaneous somatosensory stimulation activates separate pathways to DCN. (A)** Representative temporal responses of fusiform cells to transcutaneous electrical stimulation (gray dots at time 0). The absence of spikes before the onset is due to artifact removal during spike sorting. Onset latencies, rates and subsequent sustained responses (Sus) were quantified in **(B,C)**. Unimodal face/trigeminal (Tg; red) and neck/dorsal column (DCo; blue) stimulation evoked similar onset **(B)** and sustained responses **(C)**, but showed different first-spike latency (FSL) distributions (inset). Inset in **(C)**: median and quartile FSL; values above the plots represent the number of units. ^∗^*P* < 0.05.

Both face (trigeminal) and neck (dorsal column) unimodal electrical stimulation evoked responses in identified fusiform cells similar to those previously reported using deep brain stimulation (Koehler and Shore, 2013a,b). 19 of 155 (12%) units responded to face stimulation and 43 of 129 (33%) units responded to neck stimulation. **Figure 2A** shows a representative unit response to unimodal electrical stimulation of the face: a sharp onset response followed by weak sustained firing. Evoked firing rates (averaged over 20 ms after the onset response) were similar for facial and neck stimulation (Mann–Whitney *U* = 573, *P* = 0.12; **Figure 2B**). The modes of the first spike latency (FSL) distributions were 3 and 11 ms for face stimulation and 4–5 ms for neck stimulation (**Figure 2C**). The median FSLs were 12 and 5 ms, respectively (**Figure 2C**, inset). The difference in FSL distributions was significant (Kolmogorov–Smirnov test, *Z* = 0.38, *P* = 0.041), suggesting that these inputs were transmitted to the DCN via separate (trigeminal vs. dorsal column) pathways (Wright and Ryugo, 1996; Zhou and Shore, 2004; Haenggeli et al., 2005; Zeng et al., 2011).

### Paired Auditory–Somatosensory Stimulation Induces Long-term Enhancement or Suppression of Fusiform Cell Responses

**FIGURE 3:**
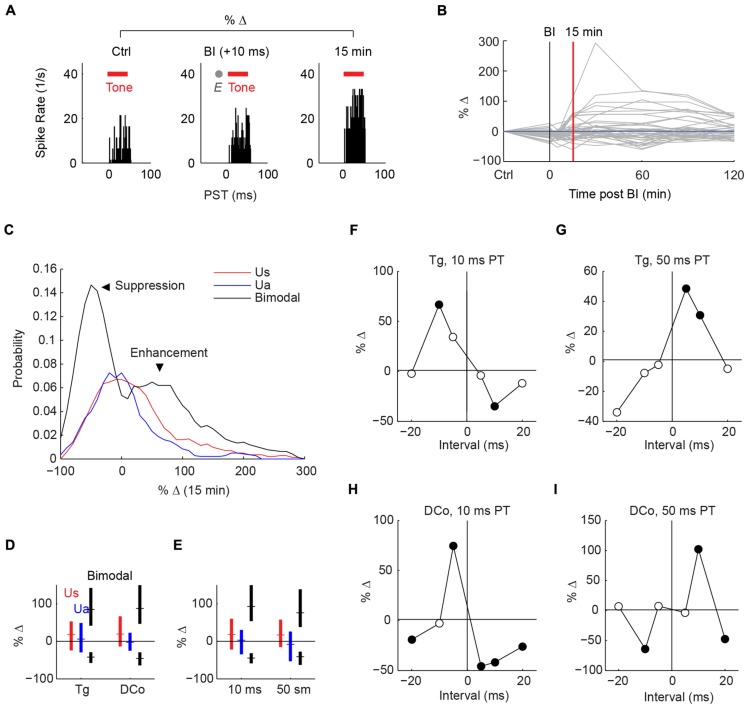
**Stimulus-timing-dependent plasticity induced by paired auditory and transcutaneous somatosensory stimulation. (A)** An example of long-lasting enhancement (15 min) induced by paired face/Tg transcutaneous and 50 ms tone stimulation at a bimodal interval (BI) of +10 ms. Plasticity is quantified as percent change in tone-evoked activity from pre- (control; Ctrl) to post-bimodal stimulation. Tone duration is denoted by red bars and electrical stimulation *(E)* by the gray dot. **(B)** Changes in tone-evoked activity from control to 0 (during pairing), 5, 15, 30, 60, 90, and 120 min after bimodal pairing. **(C)** Distribution (histogram) of percent change in tone-evoked responses from control to 15 min post-bimodal pairing. Unimodal auditory (Ua) and somatosensory (Us) stimulation produce median changes of 0, while bimodal stimulation produces enhancement (positive change) or suppression (negative change). **(D,E)** Median and quartile percent changes parsed by activation pathway: **(D)** face/ Tg or neck/dorsal column (DCo); **(E)** or pairing tone (PT) duration (10 or 50 ms). **(F–I)** Percent changes as functions of bimodal intervals, or “timing rules”, for different stimulus parameters: **(F)** face/Tg with 10 ms PTs or **(G)** 50 ms PT, **(H)** neck/DCo with 10 ms, or **(I)** 50 ms PT. Closed circles, significant changes from 0; open circles, no significance.

Studies using paired auditory and deep brain spinal trigeminal nucleus (Sp5) stimulation demonstrated long-lasting *in vivo* plasticity in fusiform cells (Dehmel et al., 2012; Koehler and Shore, 2013a,b). Plasticity induction required bimodal pairings comprised of repeated presentations of Sp5 and auditory stimuli with short temporal gaps. In the present study, Sp5 stimulation was replaced with transcutaneous activation of the trigeminal pathways. In addition, the dorsal column pathways were activated via neck stimulation. **Figure 3A** shows an example of plasticity induction. The fusiform cell response to tones 15 min after bimodal pairing was compared with the control (response to the tone alone) before bimodal pairing. In this figure, a temporal gap of 10 ms (“bimodal interval”: BI = +10 ms) produced immediate enhancement. Enhancement, or suppression in other cases, could persist for more than 120 min (**Figure 3B**). Maximum effects were observed at a median of 60 min, and recovery from plasticity at 90 min. Bimodal pairing produced greater enhancement and suppression than repeated tones or electrical stimulation alone (**Figures 3C–E**).

### Hebbian or anti-Hebbian Plasticity is Dependent on Pairing Tone Duration

**FIGURE 4:**
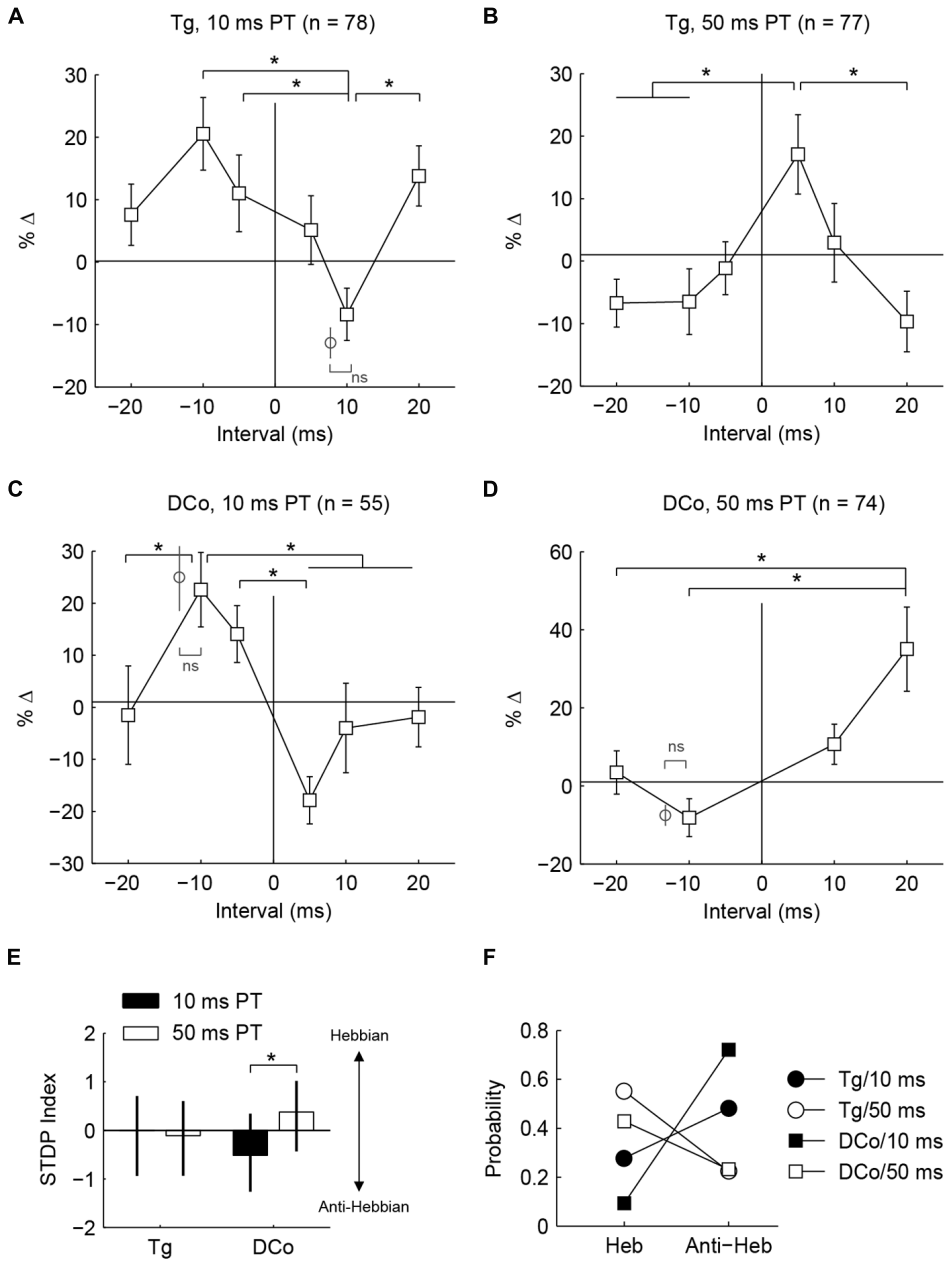
**Whether population timing rules are Hebbian or anti-Hebbian depends on PT duration. (A–D)** Percent changes in mean tone-evoked rates as functions of bimodal intervals (timing rules) corresponding to **Figures 3C–F**. Additional data points (gray circles) outside of line plots were collected in control experiments wherein a single bimodal interval was used in one experiment (ns: no significance; ^∗^*P* < 0.05) **(E)** Median and quartile STDP index for face/ Tg and neck/ DCo using 10 or 50 ms duration PTs. Index represents the difference between sums of relative changes for positive and negative bimodal intervals. A positive index indicates a Hebbian-like timing rule while a negative index indicates an anti-Hebbian-like timing rule. **(F)** Distribution of Hebbian (Heb) and anti-Hebbian (Anti-Heb) rules by stimulus parameters.

Whether fusiform cells show enhancement or suppression is dependent on the BI (Koehler and Shore, 2013a,b). In addition to varying the BI as in Koehler and Shore (2013a,b), two more variables were introduced here: stimulus location and pairing tone (PT) length (**Figure 1B**). **Figures 3F–I** display representative STDP for face (trigeminal) and neck (dorsal column) stimulation with PT durations of 10 and 50 ms. STDP indices were anti-Hebbian for units in **Figures 3F,H**, and Hebbian for units in **Figures 3G,I**. The population STDP corresponding to pairing schemes of **Figures 3F–I** is shown in **Figures 4A–D**. Experiments in which only one BI was assessed (see circles in **Figures 4A,C,D**) demonstrated that interleaved bimodal pairing protocol did not affect subsequent plasticity (*P* > 0.05 for one-BI vs. BI randomized). Face/trigeminal stimulation induced predominantly anti-Hebbian plasticity with 10 ms PT [Kruskal–Wallis test, *H*_(5)_ = 18.6, *P* = 0.0023] and Hebbian plasticity with 50 ms PTs [*H*_(5)_ = 18.5, *P* = 0.0024]. Neck/dorsal column stimulation also induced both anti-Hebbian and Hebbian plasticity that was determined by PT duration: anti-Hebbian with 10 ms PT [*H*_(5)_ = 26.2, *P* = 8.1 × 10^-5^] and Hebbian with 50 ms PT [*H*_(3)_ = 16.1, *P* = 0.0066].

To quantify population tendencies for Hebbian or anti-Hebbian plasticity, the sum of relative changes for the positive intervals (+5, +10, and +20 BIs) was subtracted from the sum of relative changes for the negative intervals (-5, -10, and -10 BIs) resulting in a Hebbian timing rule with a positive index and an anti-Hebbian timing rule with a negative index (**Figure 4E**). A larger difference with PT duration was observed for neck/dorsal column stimulation. Median and quartile STDP indices for the four different pairing schemes are plotted in **Figure 4E**. A *post hoc* analysis of ANOVA (Kruskal–Wallis test, *H*_(3)_ = 14.3, *P* = 0.003) showed a significant difference between 10 and 50 ms PTs for neck/dorsal column stimulation but not for face/trigeminal stimulation.

**FIGURE 5:**
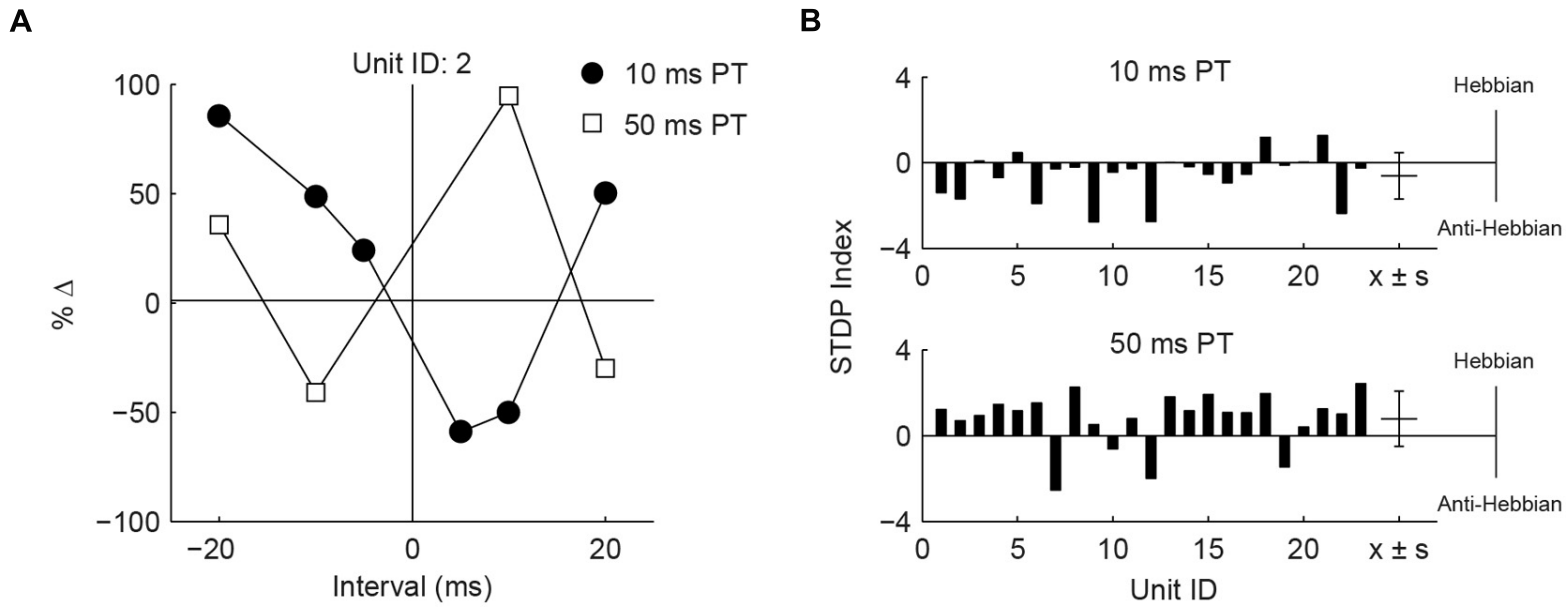
**Hebbian or anti-Hebbian timing rules can be induced in the same units using different duration PTs. (A)** Timing rules induced by neck/dorsal column stimulation paired with 10 and 50 ms PTs in a representative unit. **(B)** STDP indices of 23 units using 10 ms PT (upper panel) or 50 ms PT (lower panel). Mean ± SD (*x* ± *s*) STDP indices shown on the right.

Differences in the effects of PT duration can also be observed by counting the proportion of STDP rules produced using either the 50 or 10 ms duration (**Figure 4F**). For both face/trigeminal and neck/dorsal column stimulation, 10 ms PTs produced predominantly anti-Hebbian timing rules while 50 ms PT produced predominantly Hebbian timing rules. STDP proportions were significantly different between PT duration ($\chi_{\left(3\right)}^{2}$ = 10.5, *P* = 0.014 for trigeminal and $\chi_{\left(3\right)}^{2}$ = 26.2, *P* = 8.6 × 10^-6^ for dorsal column) but not stimulation location ($\chi_{\left(3\right)}^{2}$) = 7.7, *P* = 0.052 for 10 ms PT and $\chi_{\left(3\right)}^{2}$ = 2.9, *P* = 0.41 for 50 ms PT). To confirm the effect of PT duration, STDP curves were generated in the same units using both 10 and 50 ms PT paired with face/trigeminal stimulation (**Figure 5A**). In this instance, BIs for 10 ms PTs and 50 ms PTs were randomized (both PTs, interleaved with all BIs). 15 out of 23 units showed STDP reversals from anti-Hebbian to Hebbian when PT was changed from 10 to 50 ms (**Figure 5B**). The mean STDP index increased, as expected (Wilcoxon’s signed-rank test, *Z* = -3.3, P = 8.2 × 10^-4^).

### Fusiform Cell Tonal Responses Predict STDP Outcome

**FIGURE 6:**
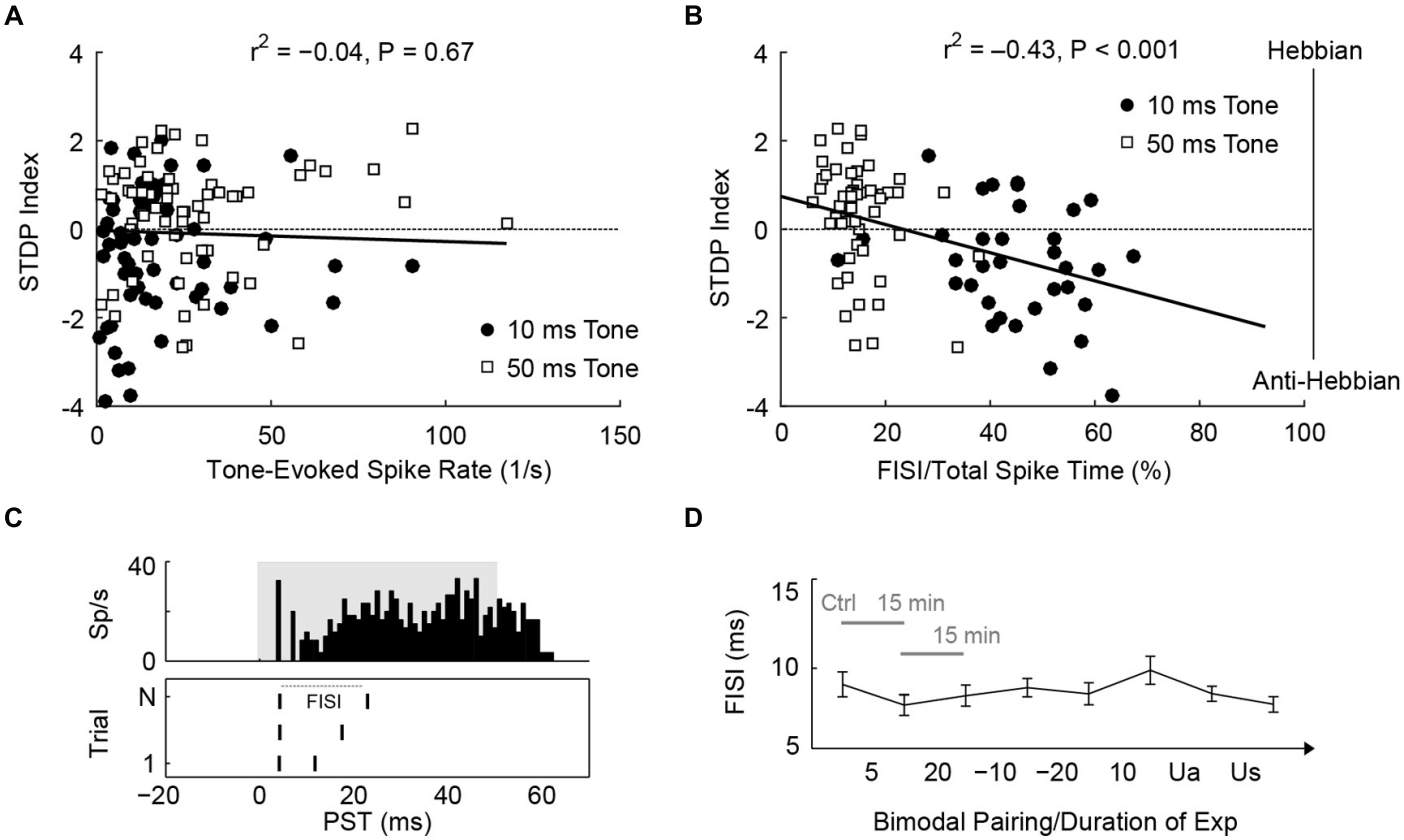
**STDP is determined by first inter-spike intervals of tone-evoked responses. (A)** STDP index does not correlate with tone-evoked spike rate. Tonal responses (10 and 50 ms) were evaluated before STDP assessment. **(B)** An example for calculating FISI in a pauser unit (upper panel; 50 ms tones at BF and 20 dB SL). The raster plot (lower panel) shows first and second spikes across trials (*N*). **(C)** Mean FISI as a percentage of total evoked spike activity correlates with STDP index. **(D)** FISIs were tracked across the duration of an STDP experiment (*n* = 30 units, paired 50 ms tones and face/Tg stimulation). The first data point was assessed before STDP induction, subsequent data points were assessed 15 min after each bimodal interval. The bimodal pairing protocol used is indicated between each data point. Ua, unimodal auditory; Us, unimodal somatosensory.

Since STDPs are largely influenced by PT duration, mechanisms that control STDP may be modulated differently by 10 or 50 ms tones. First, 50 ms tones produced higher spike counts than 10 ms tones (*U* = 11,708, *P* = 8.2 × 10^-4^). However, spike rate did not correlate with STDP outcomes [**Figure 6A**; Kendall’s tau, $r_{\left(123\right)}^{2}$ = -0.04, *P* = 0.67], suggesting that the amount of tone-evoked spikes during bimodal pairing does not affect STDP. Instead, 50 ms tones activated fusiform cells for a longer time (median duration with significant evoked spikes: 55.4 ms) than 10 ms tones (median: 15.7 ms). The duration of evoked activities, or interval with significant PT-evoked responses, indeed correlated with timing rule outcomes [$r_{\left(139\right)}^{2}$ = 0.31, *P* = 1.4 × 10^-4^]. The difference in timing rule outcome may, therefore, be due to the duration of sustained firing: fusiform cells typically show pauser or buildup temporal patterns (**Figure 6B**, upper panel), and 10 ms tones are not long enough to generate buildup phases, in which delayed first spikes or second spikes occur. Thus, we hypothesized that the buildup phases may determine STDP outcomes. To quantify the buildup phase, first inter-spike intervals (FISI) were calculated (**Figure 6B**, lower panel); a higher FISI value indicated a longer buildup phase. A significant correlation was found between the mean FISI (as a percentage of total duration of evoked activity) of each fusiform cell and STDP index [$r_{\left(85\right)}^{2}$ = -0.43, *P* = 3.5 × 10^-5^; **Figure 6C**]. STDP became anti-Hebbian as shorter tones increased the relative FISIs. To rule out possible effects of bimodal plasticity on FISI, we showed that FISI remained stable throughout the experimental duration [Kruskal–Wallis test, *H*_(7)_ = 7.5, *P* = 0.38; **Figure 6D**].

**FIGURE 7:**
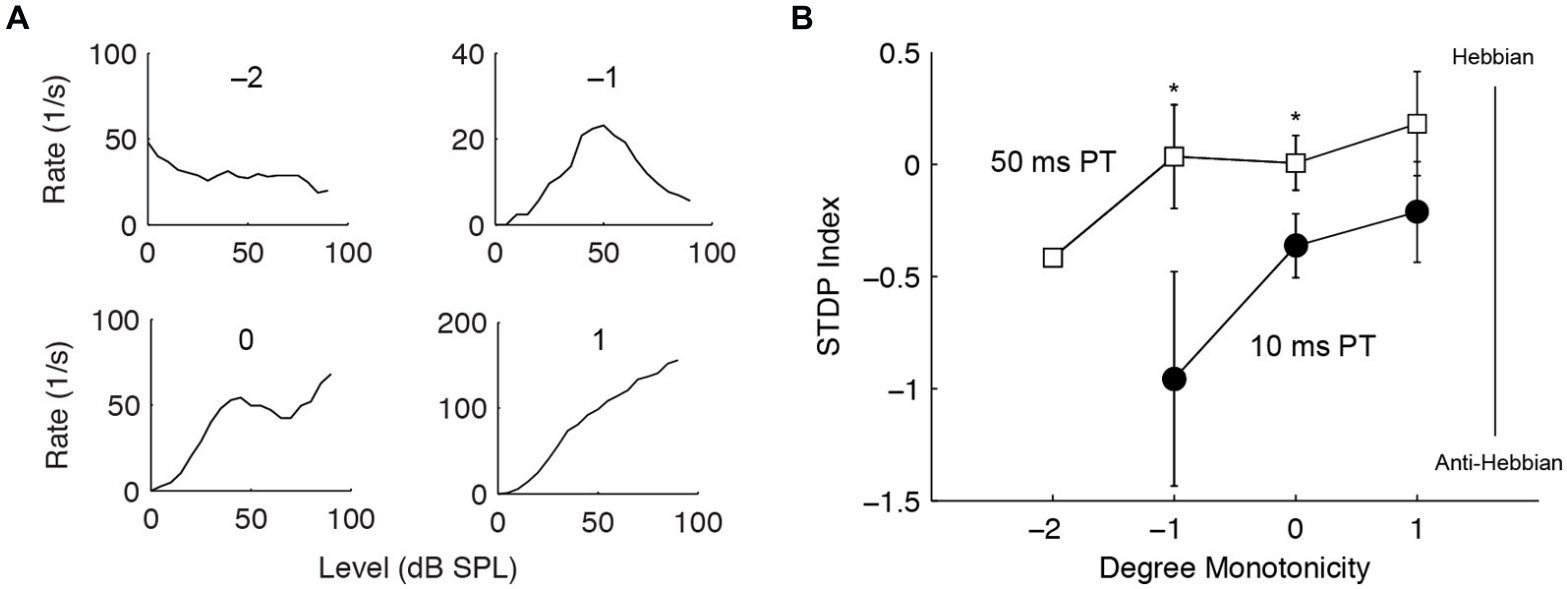
**Stronger inhibition predicts Hebbian–anti-Hebbian timing rule divergence. (A)** Four representative units’ rate-level responses showing different degrees of monotonicity. Monotonicity of -2 indicates highly non-monotonic responses (strong inhibition) while that of 1 indicates monotonic responses (weaker or no inhibition). **(B)** STDP index as a function of monotonicity. Non-monotonic (degree -2, -1, 0) and monotonic (degree 1) units that underwent both STDP induction using 10 or 50 ms PT were compared. ^∗^*P* < 0.05.

The buildup temporal pattern of fusiform cells is also influenced by inhibition (Kanold and Manis, 1999, 2001, 2005), likely derived from vertical cells in the deep layer of DCN (Rhode, 1999; Muniak and Ryugo, 2014). Thus, we tested whether units receiving stronger inhibition produce different STDP. Inhibition was identified by the degree of monotonicity in rate-level functions and categorized into four types: inhibition to below spontaneous rate (-2), highly non-monotonic (-1), weakly non-monotonic (0), and monotonic (1) (**Figure 7A**). Monotonic units showed slight trends toward higher STDP indices, or more Hebbian timing rules (**Figure 7B**); however, this was not statistically significant. Nevertheless, the difference between the 10 and the 50 ms PT-induced STDP was greater in non-monotonic units [two-way ANOVA, *F*_(2,1)_ = 5.4, *P* = 0.02 for PT duration, *P* < 0.05 for -1 and 0 degree monotonicity], confirming a role of inhibitory influence in bimodal plasticity.

## Discussion

In this study we used transcutaneous electrical stimulation to activate trigeminal and dorsal column somatosensory afferent pathways to the DCN. The response properties evoked in this manner were consistent with those described with deep brain stimulation. First, the response latencies of fusiform cells in response to face and neck stimulation were consistent with those to trigeminal ganglion (Shore et al., 2003) and cervical nerve stimulation (Kanold and Young, 2001), respectively. Both types of transcutaneous electrical stimulation produced similar, complex responses in fusiform cells (mixed excitation and inhibition; Davis and Young, 1997; Shore, 2005), suggesting activation of the shared granule cell circuit in DCN. Granule cells extend parallel fiber axons into the DCN molecular layer and synapse on the apical dendrites of fusiform and the inhibitory interneurons, cartwheel cells (Mugnaini et al., 1980; Smith and Rhode, 1985), where STDP occurs. Fusiform cells show Hebbian plasticity, while cartwheel cells show anti-Hebbian plasticity *in vitro* (Tzounopoulos et al., 2004). In the complex *in vivo* environment, however, STDP patterns across the fusiform cell population were not uniform (Koehler and Shore, 2013b). In addition, the present study showed that changing stimulus parameters can play a role in the resulting plasticity, even in the same fusiform cells. Replacing Sp5 (Koehler and Shore, 2013b) with transcutaneous facial stimulation while persevering other parameters, replicated the previous results. Stimulating a different somatosensory afferent pathway preserved the observed plasticity outcomes, while changing PT duration resulted in a consistent change in the plasticity patterns. It is interesting that the differential effect of PT duration is more apparent for neck/dorsal column stimulation, alluding to functional differences between input pathways.

### Why Does Changing PT Duration Alter STDP Plasticity Patterns?

Stimulus-timing-dependent plasticity specificity between fusiform cells and cartwheel cells *in vitro* is mediated by input-selective endocannabinoid signaling (Tzounopoulos et al., 2007; Sedlacek et al., 2011) and cholinergic modulation of the parallel fiber synapses (Zhao and Tzounopoulos, 2011). These factors likely underlie fusiform cell STDP heterogeneity *in vivo*. However, if STDP inversion from Hebbian to anti Hebbian can be induced by changing PT duration in the same cell, there must be an additional mechanism present either at the ANF-fusiform cell synapse or fusiform soma that contributes to diverse STDP patterns *in vivo*.

A possible mechanism was suggested by the predictive relationship between fusiform cell temporal patterns and STDP outcomes. The fast-inactivating, A-type potassium channels produce the characteristic pauser/buildup temporal patterns in fusiform cells as a result of delayed spiking after prolonged hyperpolarization (Manis, 1990; Kanold and Manis, 1999). A-type potassium channels also regulate signal transduction and excitability in dendrites of hippocampal pyramidal cells (Johnston et al., 2003; Frick et al., 2004), thereby likely affecting spike timing. Thus, the correlation between fusiform cell temporal responses and STDP implicates the A-type potassium channel as an underlying factor. This hypothesis also explains the observation that cells receiving stronger inhibitory influences (hyperpolarization) also show more anti-Hebbian STDP, consistent with previous findings (Koehler and Shore, 2013b). Thus, differences in intrinsic properties among fusiform cells may result in STDP heterogeneity and PT-dependent alteration. However, a key question yet to be answered is how differential modulation of A-type potassium channels affects STDP.

Alternatively, it may be that PT duration exerts differential effects on the ANF-fusiform cell synapse. This is less likely as ANF synapses on fusiform cells do not show long-term plasticity (Fujino and Oertel, 2003). However, a recent study reported that vertical cell inhibition of fusiform cells can undergo short-term facilitation (Sedlacek and Brenowitz, 2014), which can adjust the fusiform cell output. During the pairing protocol for STDP induction, repeated PT stimulation (of different duration tones) may be modulated by different short-term effects; short-term synaptic modifications can also influence long-term plasticity (Harvey-Girard et al., 2010).

### Functional Implications

Hebbian and anti-Hebbian timing underlie different neural processes. The electrosensory system of the mormyrid electric fish serves as an example of anti-Hebbian functionality (Bell, 1982; Roberts and Leen, 2010). The animal produces electrical discharges for communication, while also relying on electro-sensation for movement and navigation. To distinguish between internally generated electric fields from environmental cues, the electrosensory lobe (ELL; a structural analog of the mammalian cerebellum and DCN) receives a copy of the electrical discharge command. ELL neural activity is suppressed when the command signals (feedback) arrive prior to sensory input; sensory input that precedes command feedback is otherwise amplified. This gain adjustment via an anti-Hebbian mechanism is also evident in the mammalian cerebellum (Piochon et al., 2012). Hebbian plasticity, on the other hand, sensitizes rather than suppresses corollary stimuli. This process is important for the development of neural circuits, such as experience-dependent formation of visual fields in the optic tectum (Mu and Poo, 2006), visual cortex (Yao and Dan, 2001), or refinement of the auditory cortical tuning map (Dahmen et al., 2008). In the context of bimodal integration in DCN, anti-Hebbian plasticity likely modulates adaptive filtering. For instance, mastication produces internally generated sounds as well as orofacial inputs (via the trigeminal pathway) to DCN. When the internal feedback signal precedes auditory input, fusiform cell output is suppressed via anti-Hebbian plasticity, and perception of internally generated sound is attenuated. The dorsal column pathway, on the other hand, transmits information regarding neck motion, which changes the head-related transfer function that is detected in DCN (Oertel and Young, 2004). It is likely that internally generated alterations in sound localization cues are also suppressed via anti-Hebbian plasticity. Interestingly, if the generated sounds persist for a longer duration, the circuit would shift toward Hebbian plasticity, perhaps as a mechanism to “unlearn” or “reset” an adaptive filter and adjust circuit connectivity.

### Induction of STDP: Toward Undoing Pathological Circuitry in Tinnitus

Tinnitus, the phantom auditory perception, is correlated with fusiform cell hyperactivity (Brozoski et al., 2002; Kaltenbach et al., 2004; Roberts et al., 2010; Dehmel et al., 2012; Koehler and Shore, 2013a; Stefanescu et al., 2015). In addition, tinnitus changes auditory–somatosensory plasticity: using the same stimulus parameters (50 ms PT), normal animals showed predominant Hebbian STDP while tinnitus animals showed anti-Hebbian STDP (Koehler and Shore, 2013a). The Koehler and Shore study suggested that STDP was involved in tinnitus pathophysiology, and that fusiform cell hyperactivity is correlated with altered STDP. Thus, targeting STDP can provide a key to tinnitus treatment. In this study, we demonstrated feasibility for such a bimodal treatment strategy. STDP can be induced by applying transcutaneous electrical stimulation with tone-pairing with specific durations and bimodal intervals. Non-invasive, long-term modulation of fusiform cell activity may provide remedies for tinnitus-related pathology beginning at the level of the DCN and are currently being explored in an animal model of tinnitus.

## Author Contributions

CW, DM, and SS designed research; CW and DM performed research; CW analyzed data; CW and SS wrote the paper.

## Conflict of Interest Statement

The authors declare that the research was conducted in the absence of any commercial or financial relationships that could be construed as a potential conflict of interest.
